# The suppressive effects of miR-508-5p on the odontogenic differentiation of human dental pulp stem cells by targeting glycoprotein non-metastatic melanomal protein B

**DOI:** 10.1186/s13287-019-1146-8

**Published:** 2019-01-22

**Authors:** Fengxi Liu, Xin Wang, Yun Yang, Rongrong Hu, Wenhao Wang, Yuliang Wang

**Affiliations:** 1 Department of Oral and Maxillofacial Surgery, Yantai Affiliated Hospital of Binzhou Medical University, No 717, Jinbu Street, Muping District, Yantai, 264100 People’s Republic of China; 2 Department of Stomatology, Maternal and Child Care Service Centre of Zibo, Zibo, 255029 People’s Republic of China; 3 Department of Blood Transfusion and Clinical Central Laboratory, PLA 107th Hospital affiliated to Binzhou Medical University, Yantai, 264002 People’s Republic of China; 4 Department of Biochemistry and Molecular Biology, Binzhou Medical University, Yantai, 264003 People’s Republic of China; 5 College of Stomatology, Binzhou Medical University, Yantai, 264003 People’s Republic of China

**Keywords:** Dental pulp stem cell, miRNA, GPNMB, Odontogenesis, Differentiation

## Abstract

**Background:**

Although the involvement of glycoprotein non-metastatic melanomal protein B (GPNMB) in regulating the odontogenic differentiation of human dental pulp stem cells (hDPCs) has been identified, the underlying mechanisms are largely unknown. The purpose of this study is to investigate the effects of miR-508-5p on the GPNMB expression and the odontogenic differentiation of hDPCs.

**Methods:**

In this study, hDPCs were isolated and identified by flow cytometric analysis. Based on bioinformatics analysis, dual luciferase reporter assay was performed to verify GPNMB acting as a target of miR-508-5p. The regulatory roles of miR-508-5p in odontogenetic differentiation of hDPCs were investigated through its inhibition or overexpression (miRNA mimics and miRNA inhibitors). qRT-PCR and Western blot analysis were used to detect the expression of odontogenetic marker genes and proteins. The assays of alkaline phosphatase (ALP) activity and Alizarin Red S staining were performed to evaluate the odontogenetic phenotype.

**Results:**

We first found that the levels of miR-508-5p expression decreased gradually during odontogenesis of hDPCs, while the expressions of GPNMB were upregulated obviously. The suppressive effects of miR-508-5p on GPNMB were determined by oligonucleotide transfection in hDPCs and dual luciferase reporter assay in 293T cells. Subsequently, the significant inhibition of hDPC odontogenesis after the overexpression of miR-508-5p was observed, which is consistent with the decreased expression levels of several odontoblast-specific genes, such as dentin matrix protein 1 (DMP-1), dentin sialophosphoprotein (DSPP), and osteocalcin (OCN), as well as the decreased activity of ALP and weakened Alizarin Red S staining. Furthermore, ectopic expression of GPNMB (lacking 3′-UTR) rescued the effects of miR-508-5p on odontogenic differentiation.

**Conclusions:**

Our study demonstrated that miR-508-5p regulated the osteogenesis of hDPCs by targeting GPNMB and provided novel insight into the critical roles of microRNAs in hDPC differentiation.

## Background

Cell-based tissue engineering has become increasingly important in regenerative medicine. Since the discovery by Gronthos et al., human dental pulp stem cells (hDPCs) have been receiving more attention recently because of its some advantages, such as easy availability, minimal invasion, multipotent ability, less immune rejection, and avoidance of ethical concerns [1–3]. These outstanding features make the hDPCs as a suitable source of tissue repair, not only in dentinogenesis but also in regeneration-related diseases [4, 5]. Although tremendous advances about hDPCs have been achieved in the past decade, the understandings of hDPCs are still insufficient and its regulation mechanism is still not clear.

MicroRNAs (miRNAs), a class of small non-coding RNAs that approximately 17 to 25 nucleotides in length, can regulate gene expression by recognizing the 3′ untranslated region (3′-UTR) of their target mRNAs leading to mRNA decay or translation repression [6]. It has been demonstrated that miRNAs play essential roles in a broad range of biological processes, including development, cell proliferation/apoptosis, signal pathways, inflammation, and tumors [7–9]. Similarly, miRNAs have emerged as important regulators in stem cell biology, related to cell reprogramming, maintenance of stemness, and regulation of cell differentiation [10, 11]. Recently, although the role of miRNAs in the proliferation and differentiation of hDPCs has attracted more attention, the limited understanding of the molecular mechanism underlying their differentiation is also a huge obstacle prior to the usage of hDPCs in cell therapy.

Glycoprotein non-metastatic melanomal protein B (GPNMB), also known as osteoactivin, is a type 1 transmembrane glycoprotein and expressed in a wide array of tissues and plays regulatory roles in various cellular functions, including cell adhesion, migration, and differentiation [12]. Thus, GPNMB has been implicated in physiological and pathophysiological cascades of tissue injury and repair. Since the first report that GPNMB played an essential role in osteoblast differentiation and matrix mineralization, increasing evidence suggested that GPNMB can served as an osteoblast-specific gene in the bone and play a vital role for osteoblast differentiation [13, 14]. As the great similarity between osteogenic and odontogenic differentiation, our findings and other published studies have also demonstrated the involvement of GPNMB in regulating the odontogenic differentiation of hDPCs [15]. However, the mechanisms involving the regulation of GPNMB during the odontogenic differentiation of hDPCs are still unclear.

In this study, we tested the hypothesis that the changes of GPNMB expression can be regulated in the miRNA level during the odontogenesis of hDPCs. Bioinformatics analysis was first performed to reveal miRNA 508-5p (miR-508-5p) as a potential regulator for GPNMB. The level of miR-508-5p and GPNMB was detected during the odontogenic differentiation of hDPCs and a negative correlation between miR-508-5p and GPNMB was observed. The luciferase reporter assays further confirmed that GPNMB is a direct target of miR-508-5p. Also, our results showed that the overexpression and knockdown of miR-508-5p can influence the differentiation capacity of hDPCs remarkably followed by the expression changes of GPNMB. Furthermore, the transfection of GPNMB without 3′-UTR reversed the effects of miR-508-5p on odontogenesis of hDPCs. These results provided evidence that miR-508-5p can inhibit the odontogenic differentiation of hDPCs by targeting GPNMB.

## Materials and methods

### Isolation and culture of hDPCs

All experimental procedures were approved by the Ethics Committee of the School of Stomatology, Binzhou Medical University. hDPCs were isolated and cultured according to our published work [15]. Briefly, extracted healthy human third molars (*n* = 5) were collected from patients who were undergoing treatment at the Dental Department of the Yantai Affiliated Hospital of Binzhou Medical University. After gentle separation from the pulp chambers, the pulp tissues were minced into tiny pieces (about 1 mm) and then digested with a solution of 3 mg/mL collagenase type I and 4 mg/mL dispase II for 1 h at 37 °C. The cell suspension was seeded in a 35-mm culture dish (Corning Costar, USA) and cultured in maintenance medium containing Dulbecco modified Eagle medium (DMEM) supplemented with 10% fetal bovine serum (FBS) and 100 U/mL penicillin/streptomycin (Sigma, St. Louis, MO, USA) at 37 °C in a humidified atmosphere of 5% CO_2_. The fresh medium was changed every 2 days. Approximately 10 days after seeding, the cells became nearly confluent with the cell number about 2–3 × 10^5^ (passage 0). The cell population was detached and transferred to 6-well plates (passage 1) (Corning Costar, USA). Cells were passaged at the ratio of 1:3 when they reached 75–85% confluence. Cells from the third passage (P3) were used for all experiments.

The multipotent differentiation potential of the hDPCs was identified by osteogenic and adipogenic differentiation induction. Briefly, the cells were exposed to osteogenic medium (DMEM supplemented with 10 nmol/L dexamethasone, 10 mmol/L β-glycerophosphate, 50 μg/mL ascorbate phosphate, 10 nmol/L 1,25-dihydroxyvitamin D_3_, and 10% FBS) and adipogenic medium (DMEM supplemented with 1 mM dexamethasone, 10 mg/mL insulin, 100 mM indomethacin, 0.5 mM methylisobutylzanthine, and 10% FBS) for 14 days. Alizarin Red S and Oil Red O reagent were used to visualize the calcium accumulation and oil droplets, respectively. After staining, the cells were imaged by an inverted light microscope (Olympus, Japan).

For odontoblastic induction, hDPCs were plated in 6-well plates (Corning Costar, USA) at an initial density of 2 × 10^5^ cells/well and cultured in DMEM supplemented with 10% FBS, antibiotics, 50 mg/mL ascorbic acid, 10 mmol/L sodium β-glycerophosphate, and 10 nmol/L dexamethasone (Sigma, St. Louis, MO, USA) for 14 days. The culture medium was changed every 3 days.

### Flow cytometric analysis of hDPCs

hDPCs (P3) were characterized by fluorescence-activated cell sorting (FACS) (BD Bioscience, Franklin Lake, NJ). Cells were incubated with the FITC-conjugated mouse monoclonal antibody against mouse CD29 (Cat # 11-0299-42), CD34 (Cat # 11-0349-42), CD45 (Cat # MHCD4530TR), CD90 (Cat # 11-0909-42), and CD105 (Cat # MA5-11854). All antibodies were purchased from Invitrogen. Isotype-identical antibodies served as controls. The number of positive cells in 1 × 10^5^ was determined using FACSuite software (BD Bioscience).

### miRNA target prediction

Prediction of miRNA target genes was performed using the TargetScan (http://www.targetscan.org/) and PicTar (http://www.pictar.org) databases.

### Dual luciferase reporter assay

A GPNMB 3′-UTR reporter vector was synthesized by Yingrun Biotechnology (China). Either wild-type GPNMB or its mutant fragment was inserted in the vectors (Table 1) and was termed GPNMB-Wt and GPNMB-Mt, respectively. The sequences of the miR-508-5p binding site and the mutant site are underlined.

**Table 1:** Sequences used in the dual luciferase reporter assay

Name	Sequence (5′-3′)
GPNMB-3′-UTR-Wt	……CAAGAATTTAAAGGAGTTTCTTAAATTTCGACCTTGTTTCTGAAGCTCACTTTTCAGTGCCATTGATGTGAGATGTGCT*GGA*GTGGCTATTAACCTTTTTTTCCTAAAGATTATTGTTAAATAGATATTGTGGTTTGGGGAAGTTGAATTTTTTATAGGTTAAAT……
GPNMB-3′-UTR-Mt	……CAAGAATTTAAAGGAGTTTCTTAAATTTCGACCTTGTTTCTGAAGCTCACTTTTCAGTGCCATTGATGTGAGATGTGCT*CCT*GTGGCTATTAACCTTTTTTTCCTAAAGATTATTGTTAAATAGATATTGTGGTTTGGGGAAGTTGAATTTTTTATAGGTTAAAT……

The italicized letters indicate the different nucleotieds between GPNMB-3'-UTR-Wt and GPNMB-3'-UTR-Mut.

To assess the effects of miR-508-5p on the expression of GPNMB in hDPCs, cells were transfected with several oligonucleotides, including miR-508-5p-negative controls (NC, 50 nM), negative controls for miR-508-5p siRNA (NC-Si, 50 nM), miR-508-5p mimics (50 nM), or miR-508-5p inhibitors (siRNA, 50 nM). All oligonucleotides were purchased from RiboBio (Guangzhou, China). Transfection of miRNAs was carried out using Lipofectamine 2000 according to the manufacturer’s procedure (Invitrogen, USA). Cells were harvested after 48 h for the analysis of GPNMB expression.

To estimate whether miR-508-5p could bind to the GPNMB 3′-UTR, 293T cells were seeded in 96-well plates (Corning Costar, USA). The cells were transfected with the GPNMB-Wt or GPNMB-Mt reporter plasmid, miR-508-5p-negative controls (NC, 50 nM), negative controls for miR-508-5p siRNA (NC-Si, 50 nM), miR-508-5p mimics (50 nM), or miR-508-5p inhibitors (siRNA, 50 nM) (RiboBio, Guangzhou, China) using Lipofectamine 2000 (Invitrogen, USA). According to the protocol of the manufacturer, cells were harvested after 48 h, and then firefly and Renilla luciferase activities were assayed using the dual luciferase reporter assay system (Promega, USA). Renilla luciferase activity was normalized to firefly luciferase activity.

### Alkaline phosphatase activity analysis

Briefly, hDPC cells from each group were grown in odonto-induction media for 7 days. Alkaline phosphatase (ALP) activity was determined using an ALP assay kit (Sigma, St. Louis, MO, USA) according to the manufacturer’s protocol. The absorbance was detected in the microplate reader at 520 nm wavelength. The protein content was quantified using a BCA protein assay (Beyotime, Haimen, China). ALP activity was normalized to the total protein content.

### Alizarin Red S staining

For Alizarin Red S staining, hDPC cells from each group grown in odonto-induction media for 14 days were washed with PBS and fixed in ice-cold 70% ethanol for 1 h. After several rinsing with deionized water, 2% Alizarin Red S was added to the cells (15 min incubation at room temperature). After several rinsing with deionized water, orange-red calcium nodules were observed using an inverted light microscope (Olympus, Japan).

### Total RNA isolation and qRT-PCR analysis

Total RNA was extracted from each cell sample using TRIzol (Invitrogen, USA), and cDNA was synthesized using a PrimeScript RT reagent kit (Takara, Dalian, China) according to the manufacturer’s protocol. Quantitative real-time polymerase chain reaction was performed using Premix Ex Taq (Takara, Dalian, China) on Bio-Rad iQ5 Quantitative PCR System (Takara Bio Inc., Otsu, Japan). The levels of mRNA transcripts were analyzed by using the specific primers and SYBR Green I reagent according to the manufacturer’s instructions. For the analysis of mRNA levels, GAPDH was used as an endogenous normalization control. For miR-508-5p expression analysis, the level of miRNA was evaluated through the normalization to that of the internal control U6. The primer sequences of the evaluated genes are listed in Table 2.

**Table 2:** Primers for real-time RT-PCR

Gene	Accession	Forward	Reverse
*GPNMB*	NM_001005340.1	AAGTGAAAGATGTGTACGTGGTAACAG	TCGGATGAATTTCGATCGTTCT
*ALP*	NM_000478.5	CTATCCTGGCTCCGTGCTC	GCTGGCAGTGGTCAGATGTT
*DSPP*	NM_014208.3	GGGACACAGGAAAAGCAGAA	TGCTCCATTCCCACTAGGAC
*DMP-1*	NM_004407.3	GTGAGTGAGTCCAGGGGAGATAA	TTTTGAGTGGGAGAGTGTGTGCC
*OCN*	NM_001199662.1	CTCACACTCCTCGCCCTATT	TTGGACACAAAGGCTGCAC
*GAPDH*	NM_002046.3	GTTGTCTCCTGCGACTTCA	GGTGGTCCAGGGTTTCTTA
*miR-508-5p*	MI0003195	ACACTCCAGCTGGGTACTCCA GAGGGCGTCACT	TGGTGTCGTGGAGTCG
*U6*	NR_004394	GCTTCGGCAGCACATATACTAAAAT	CGCTTCACGAATTTGCGTGTCAT

### Western blot

Cells were lysed, and proteins were extracted in RIPA buffer (Bioteke, Beijing, China), containing 25 mM Tris-HCl (pH 7.6), 150 mM NaCl, 1% Nonidet P-40, 1% sodium deoxycholate, 0.1% SDS, and protease inhibitor mixture. After the protein concentration determined by BCA, the mixture of total protein was separated by 10% SDS-PAGE electrophoresis, and then the proteins were transferred to PVDF membrane (Amersham, Little Chalfont, UK). The membranes were blocked at room temperature with 5% non-fat dry milk in Tris-buffered saline (TBS) for 1 h followed by incubation in the primary antibodies (anti-GPNMB, Cat # MA5-24014; anti-DSPP, Cat # PA5-72040; anti-DMP-1, Cat # PA5-19009; anti-OCN, Cat # MA1-20788; anti-GAPDH, Cat # A21994 from Invitrogen) overnight at 4 °C. After being washed for 5 min, the membranes were incubated with secondary antibodies conjugated to horseradish peroxidase (Cat # 31430, Invitrogen) for detection. The air-dried membranes were imaged using an image analyzer. All experiments were repeated three times.

### Statistical analysis

All results are reported as means ± SD. Statistical analyses were made by the Student *t* test for a single comparison or one-way analysis of variance followed by the *Bonferroni* correction for multiple comparisons using SPSS version 14.0.1 for Windows (SPSS). Values of *p* < 0.05 were considered statistically significant.

## Results

### Characteristics of hDPCs

**Fig. 1:**
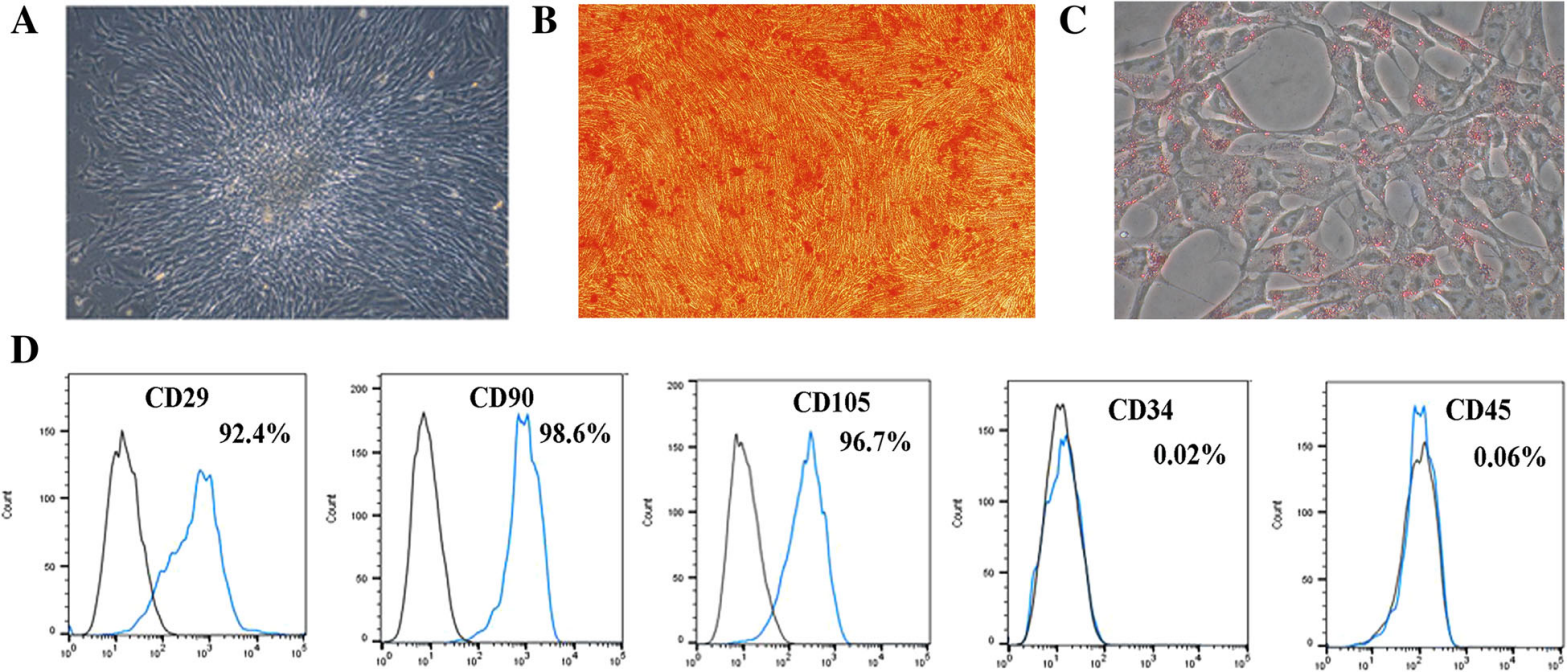
Culture and identification of hDPCs. **a** Cultured hDPCs. **b** Osteogenic differentiation of hDPCs was assessed by Alizarin Red S staining (× 50). **c** Adipogenic differentiation of hDPCs was assessed by Oil Red O stain (× 200). **d** Flow cytometric analysis revealed the surface markers of hDPCs. Cells were incubated with fluorescence-conjugated antibodies against CD29, CD90, CD105, CD34, and CD45. Isotype-identical antibodies served as controls (black line). Analysis of molecular surface antigen markers in hDPCs by flow cytometry indicated that the cells were positive for CD29, CD90, and CD105, whereas they were negative for CD34 and CD45

After about 10 days in culture, primary hDPCs extracted from enzyme digestion were round shaped, formed adherent colonies which adhered to culture dishes strongly, and displayed fibroblast-like morphology obviously (Fig. 1a). After 14 days of culture with the osteogenic differentiation, hDPCs (P3) were differentiated into osteoblasts stained with Alizarin Red S (Fig. 1b). Also, Oil Red O staining showed the accumulation of oil droplets in hDPCs (P3) subsequent to the adipogenic induction for 14 days (Fig. 1c). The surface markers of hDPCs were analyzed using flow cytometry. It has been observed that hDPCs expressed a high level of CD29, CD90, and CD105, which were common stem cell markers in mesenchymal stem cells and dental pulp stem cells. In contrast, hDPCs exhibited weak expression of CD34 and CD45 (Fig. 1d).

### miR-508-5p is downregulated during the odontogenic differentiation of hDPCs

**Fig. 2:**
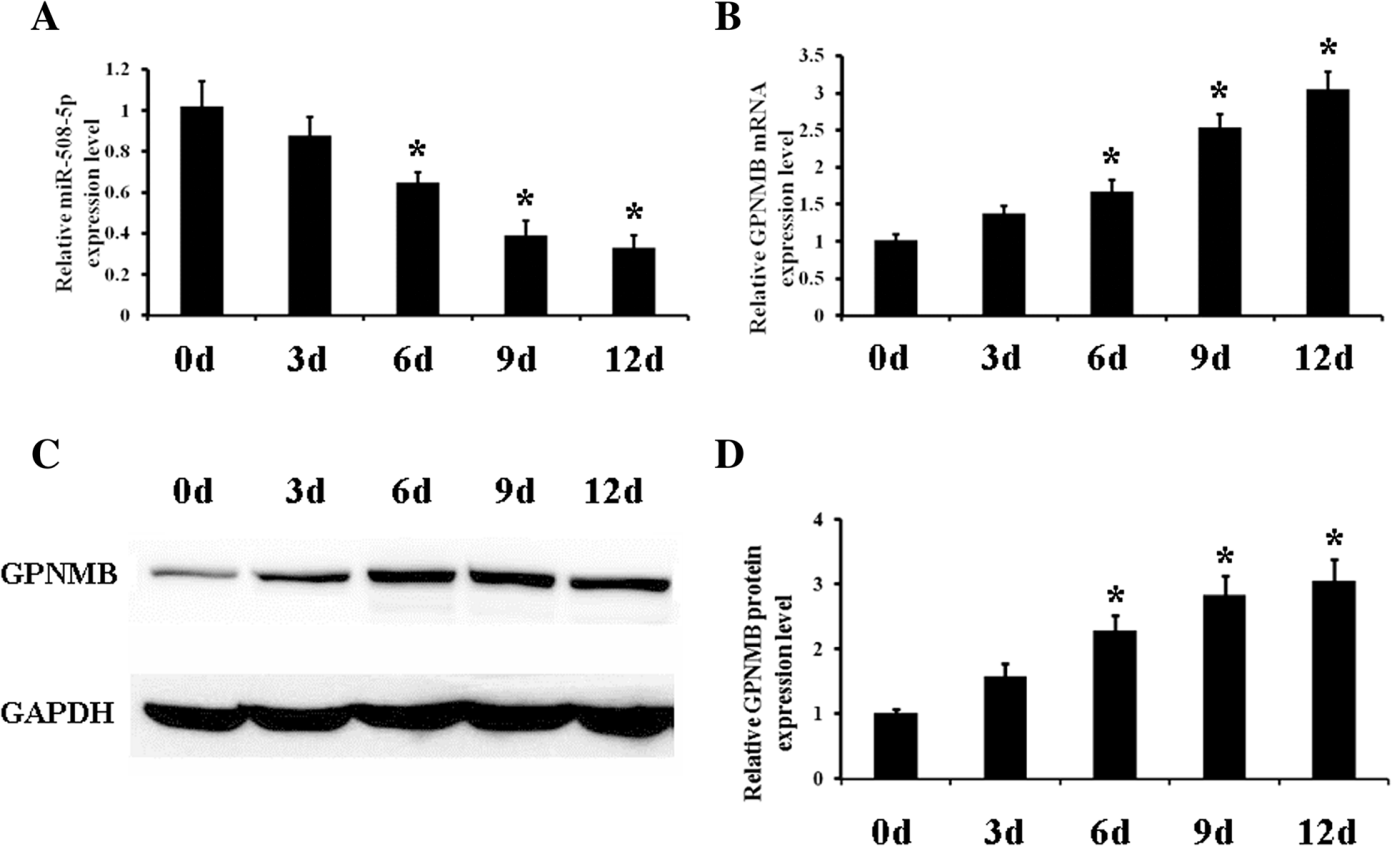
The expressions of miR-508-5p and GPNMB during odontogenic differentiation of hDPCs. **a** miR-508-5p expression levels were detected by qRT-PCR during the odontogenic differentiation of hDPCs at indicated time points. **b** The expression levels of GPNMB were detected by qRT-PCR during the odontogenic differentiation of hDPCs at indicated time points. **c** The protein levels of GPNMB were detected by Western blotting during the odontogenic differentiation of hDPCs at indicated time points. **d** Quantitative analyses of Western blot revealed GPNMB levels increased during the odontogenic differentiation of hDPCs at indicated time points. *p < 0.05 vs the group of 0d

To investigate the effect of miR-508-5p on hDPCs characteristics, we first examined the dynamic expression profile of miR-508-5p in the odontogenic differentiation of hDPCs. The results of qPCR indicated that miR-508-5p expression was significantly decreased at different time points during odontogenic differentiation (Fig. 2a). In contrast, the levels of GPNMB mRNA and protein expression were significantly upregulated when cells were cultured under odontogenic induction condition, which was consistent with our previous study (Fig. 2b–d). These findings suggested that there may have an inverse correlation between miR-508-5p and GPNMB expressions during odontogenesis of hDPCs, indicating that miR-508-5p may be a regulator of GPNMB.

### The expression of GPNMB is suppressed by miR-508-5p

**Fig. 3:**
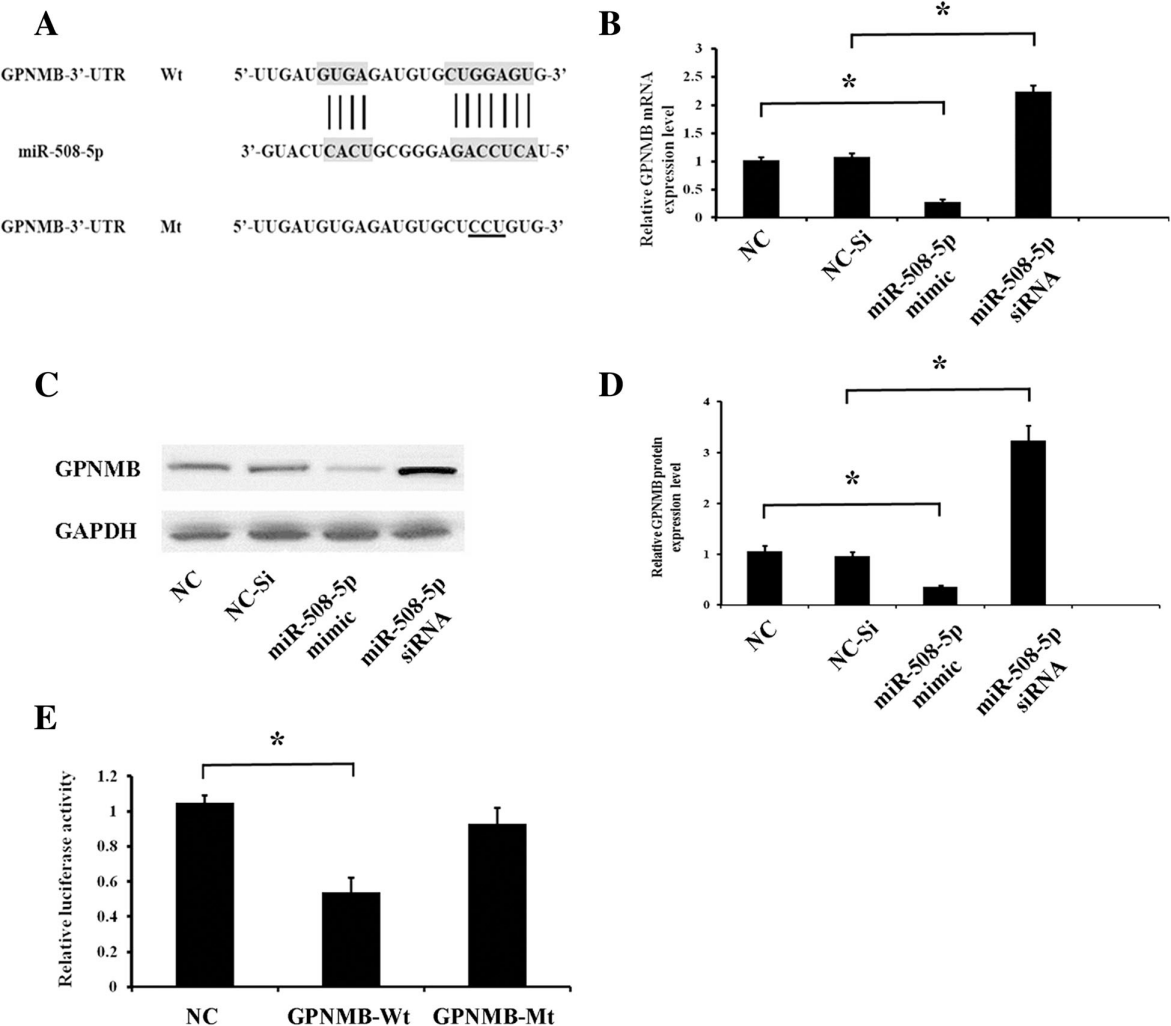
The 3′-UTR of GPNMB is a direct target of miR-508-5p. **a** Schematic illustration of the complementary sequence between miR-508-5p and the GPNMB 3′-UTR. The mutation sites on the 3′-UTR of GPNMB were underlined. **b** hDPCs were transfected with miR-508-5p mimics or siRNA, and the expression levels of GPNMB were determined by qRT-PCR on day 6. **c** The expression levels of GPNMB were detected by Western blotting after transfection of hDPCs on day 6. **d** The Western blot results of GPNMB protein were quantified. **e** Luciferase reporter assay data found that co-transfection of 293T cells with miR-508-5p and wild-type GPNMB 3′-UTR led to a decrease in luciferase activity. In contrast, there was no significant difference observed in the GPNMB-Mt group. *p < 0.05

To determine whether there is a potential link between the expression levels of GPNMB and miR-508-5p during odontogenesis of hDPCs, bioinformatics analysis was performed to search for potential targets of miR-508-5p from TargetScan and PicTar databases. The predicted results showed that there were partial complementary sequences between the 3′-UTR of GPNMB and miR-508-5p. Therefore, GPNMB may be a potential target of miR-508-5p (Fig. 3a).

Subsequently, miR-508-5p was overexpressed or silenced by transfection of hDPCs with miR-508-5p mimics or miR-508-5p siRNA to investigate whether miR-508-5p could alter the expression of GPNMB. It has been demonstrated that GPNMB can be significantly downregulated by miR-508-5p overexpression at both mRNA and protein levels (Fig. 3b–d). Conversely, the inhibition of miR-508-5p could increase the GPNMB mRNA and protein contents (Fig. 3b–d). These data further suggested that GPNMB is a potential target of miR-508-5p.

According to the prediction of TargetScan and PicTar that the 3′-UTR of GPNMB is partially complementary to miR-508-5p (Fig. 3a), we hypothesize that miR-508-5p can bind to the 3′-UTR of the GPNMB gene, thus inhibits the expression of GPNMB. To verify this hypothesis, the dual luciferase reporter assay was performed. The luciferase reporter constructs generated with the wild-type (Wt) and mutant (Mt) 3′-UTRs of GPNMB (Fig. 3a) were co-transfected into hDPCs with miR-508-5p mimics or negative control (NC). The dual luciferase activity assay detected that the relative luciferase activity of the GPNMB-Wt group decreased compared with the NC group, but the GPNMB-Mt group seemed to have hardly any difference compared to the NC group regarding luciferase activity (Fig. 3e). These results confirmed that GPNMB is a direct target of miR-508-5p, and its expression can be directly inhibited by miR-508-5p.

### miR-508-5p inhibits odontogenic differentiation of hDPCs

**Fig. 4:**
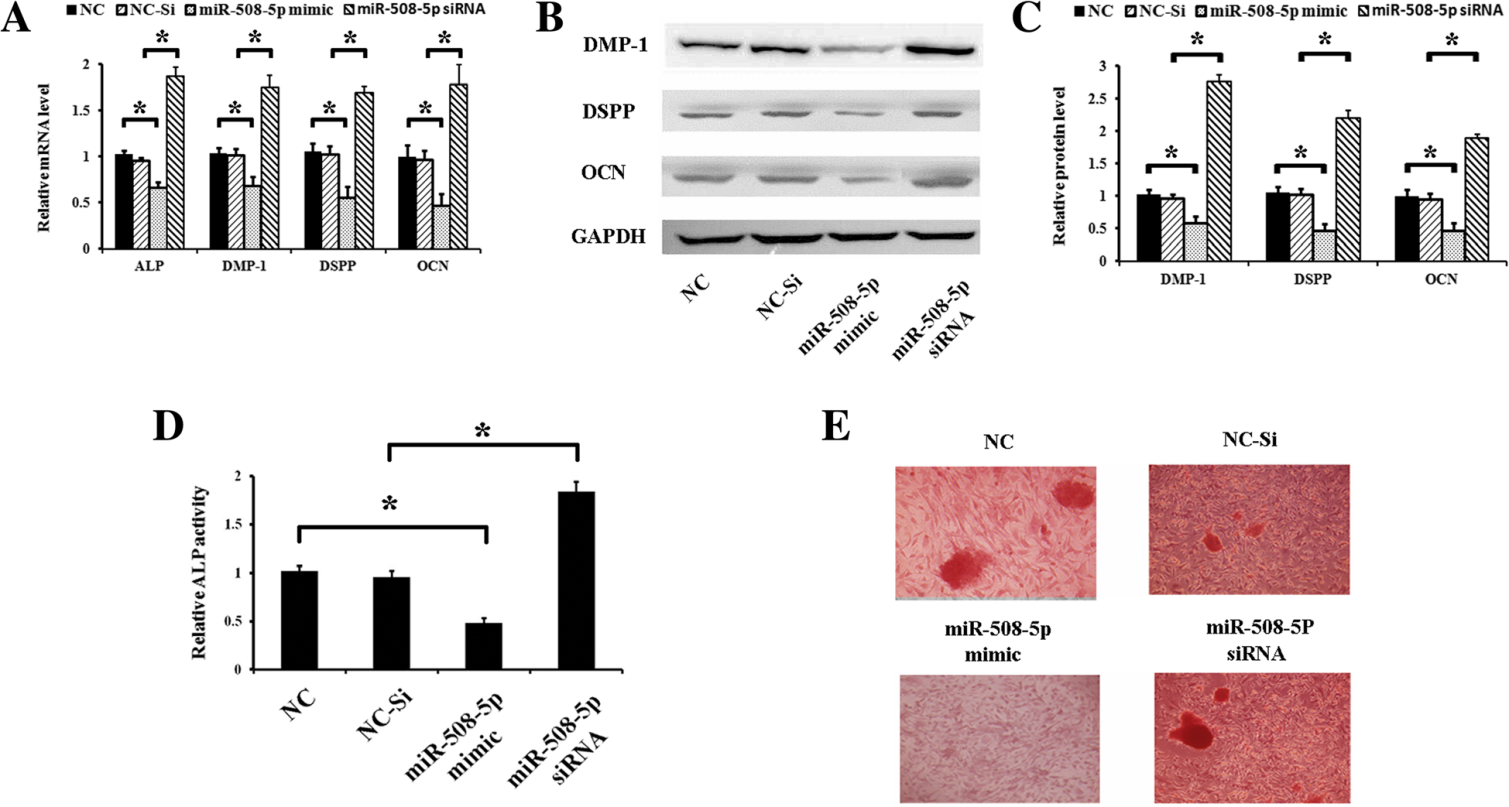
miR-508-5p inhibits the odontogenic differentiation of hDPCs. hDPCs were transfected with negative control (NC), negative control of siRNA (NC-Si), miR-508-5p mimics, or miR-508-5p siRNA, respectively. **a** On day 7 of odontogenic differentiation, the expression levels of the odontogenic marker genes (*ALP*, *DMP-1*, *DSPP*, and *OCN*) were detected by qRT-PCR. **b** The expression levels of the odontogenic marker genes (*ALP*, *DMP-1*, *DSPP*, and *OCN*) were detected by Western blotting on day 7 of odontogenic differentiation. **c** The Western blot results of GPNMB protein were quantified. **d** ALP activity decreased in the miR-508-5p mimics group and increased in the miR-508-5p siRNA on day 7 of odontogenic induction. **e** On day 14 after treatment, Alizarin Red S staining was performed to show the inhibitory effects of miR-508-5p mimics on the matrix mineralization of hDPCs. *p < 0.05

Because miR-508-5p has an inhibitory effect on GPNMB expression and our previous study showed that GPNMB could promote the differentiation of hDPCs, we determined whether miR-508-5p can regulate the odontogenic differentiation of hDPCs. hDPCs were transfected with NC, NC-Si, miR-508-5p mimics, or miR-508-5p siRNA. After the induction for 7 days, the expression of odontogenesis-related genes was assessed. The results showed that miR-508-5p overexpression significantly downregulated odontogenic differentiation, which was indicated by lower expression of the odontogenic marker genes, such as *ALP*, *DMP-1*, *DSPP*, and *OCN* (Fig. 4a–c), and decreased ALP activity (Fig. 4d) compared with cells transfected with NC group. Similarly, the reduced matrix mineralization visualized by Alizarin Red S staining was also observed after 14 days of induction (Fig. 4e). By contrast, odontogenic marker gene expression, ALP activity, and matrix mineralization were enhanced in miR-508-5p-inhibitor-treated hDPCs compared to NC-treated cells (Fig. 4a–e). These data clearly illustrate that miR-508-5p is a negative regulator of odontogenic differentiation of hDPCs.

### miR-508-5p suppresses the odontogenic differentiation of hDPCs by targeting GPNMB

**Fig. 5:**
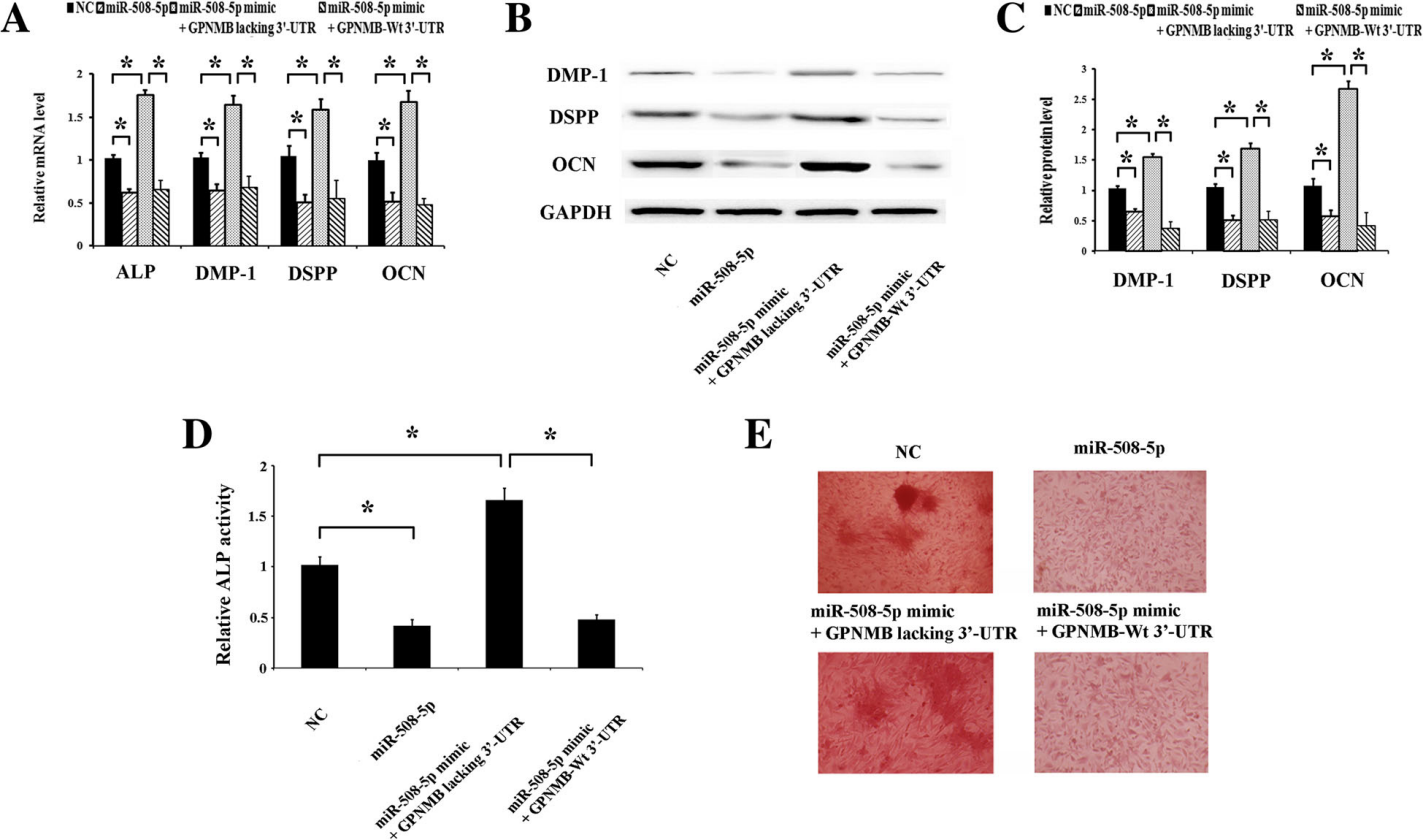
GPNMB lacking 3′-UTR can rescue the effect of miR-508-5p. **a** The expression levels of the odontogenic marker genes (*ALP*, *DMP-1*, *DSPP*, and *OCN*) were detected by qRT-PCR in each indicated group. **b** The expression levels of the odontogenic marker genes (*ALP*, *DMP-1*, *DSPP*, and *OCN*) were detected by Western blotting in each indicated group. **c** The Western blot results of GPNMB protein were quantified. **d** On day 7 after the co-transfections of miR-508-5p mimics and GPNMB lacking 3′-UTR of hDPCs, ALP activity increased significantly. **e** On day 14 after treatment, Alizarin Red S staining was performed to show the effects on the matrix mineralization of hDPCs in each indicated group. *p < 0.05

Based on the above results, speculation could be made that miR-508-5p should have some relationship with GPNMB during odontogenic differentiation. To verify the hypothesis, hDPCs were co-transfected with NC or miR-508-5p mimics along with GPNMB plasmid lacking 3′-UTR or containing wild-type 3′-UTR. The results showed that, after co-transfected with miR-508-5p mimics with GPNMB (lacking 3′-UTR), the expressions of odontogenic marker genes, such as *ALP*, *DMP-1*, *DSPP*, and *OCN*, were increased significantly (Fig. 5a–c). Similar results were observed for the assessment of ALP activity (Fig. 5d) and Alizarin Red S staining (Fig. 5e). However, co-transfection of miR-508-5p mimics and GPNMB containing the 3′-UTR sequence did not reverse the effects of miR-508-5p mimics (Fig. 5a–e). These results suggest that miR-508-5p inhibits the odontogenic differentiation of hDPCs by downregulating its target GPNMB.

## Discussion

Due to recent advances in stem cell biology and stomatology, hDPCs were identified as neural crest-derived mesenchymal progenitors [16]. Numerous studies have revealed that hDPCs have essential functions not only for tooth development in vivo but also for multipotent differentiation in vitro [17, 18]. Although it has reached a consensus that hDPCs can be used as a suitable seed cell for tissue regeneration, the clinical application of hDPCs has been severely limited by the lack of understanding of the molecular mechanism that underlies their differentiation.

GPNMB is expressed in many tissues with regulatory roles in tissue injury and repair. Also, it has been demonstrated that GPNMB plays a role in positive regulations of the viability, proliferation, and migration of MSCs [19]. To our knowledge, the investigations about GPNMB and MSCs mainly focus on its effect on osteogenic differentiation in vitro and bone formation in vivo. Abdelmagid et al. reported that GPNMB acts as a downstream mediator of BMP-2 and has significant effects on osteoblast function [20]. In human bone marrow stromal cells, the potential of differentiation into osteoblasts can be stimulated obviously in a dose-dependent manner [21]. The fact that GPNMB serves as a positive regulator of osteogenesis was further verified in a mouse model with a GPNMB mutation [22]. In contrast, studies about the function of GPNMB in odontogenic differentiation, which has great significance for proper tooth development and dental pulp regeneration, are very limited. Our previous study showed that GPNMB could inhibit the proliferation and promote the differentiation of hDPCs in vitro [15]. Similarly, in C3H10T1/2 mesenchymal stem cell (MSC) line, Arosarena et al. reported that the odontogenic differentiation of C3H10T1/2 increased with the treatment of GPNMB [23]. These results provide conclusive evidence of the involvement of GPNMB in the fate commitment of MSCs. In the meanwhile, a rationale issue needing further investigation was raised on how the expression of GPNMB was appropriately regulated during hDPCs differentiation.

As an important regulatory factor, miRNAs have received increasing attention recently. Compared with other types of stem cells, the role of microRNA in dental pulp stem cells is poorly understood. Despite this, findings from several studies took new insights into an intricate network about hDPC differentiation in the miRNA level [24–26]. It was documented that the downregulation of miR-145 and miR-143 can stimulate the odontoblast differentiation of mouse dental pulp stem cell through the promotion of dentin sialophosphoprotein (DSPP) and DMP-1 expression [27]. Also, the impact of miR-433 on the proliferative and mineralization abilities as well as cell death of dental pulp cells has been reported, which was proposed to contribute to dental pulp repair and regeneration [28]. In dental papillary cells, Li et al. observed that miR-3065-5p promoted odontoblastic differentiation through directly targeting Bmpr2 in late odontoblast differentiation [29].

In the present study, we first found that GPNMB can be regulated by miR-508-5p which was confirmed by luciferase reporter assays. The other intriguing finding is, during the odontogenetic differentiation of hDPCs, the suppressive roles of miR-508-5p on GPNMB can also be detected. These data strongly support that miR-508-5p served as a critical modulator, through the target of GPNMB, to affect the biological characteristics of hDPCs. As mentioned above, owing to the crucial role of GPNMB in tissue repair, increasing studies have been carried out to investigate the effects of GPNMB on the differentiation of stem cells and tissue regeneration. To date, it is well known that GPNMB can influence serial signal pathways, such as BMP-2 and TGF-β, both of which would be of great importance in the fields of cell functions [20, 22]. It can be anticipated that the changes of these signal cascades further trigger the induction or inhibition of gene downstream related to cell differentiation.

During the odonto/osteogenic differentiation and extracellular matrix mineralization, some specific genes, including ALP, DSPP, DMP-1, and OCN, were regarded as odontoblastic/osteoblastic markers [30, 31]. Among these markers, several mineralized matrix proteins, such as ALP, DMP-1, and OCN, were commonly used to determine the differentiation potential towards the odontogenic and/or osteogenic lineage owing to the similarity between both processes. In contrast, DSPP is considered as a more specific marker involved in the odontogenic differentiation of DPSCs [19]. Considering the remarkable influence of miR-508-5p on the odontogenic differentiation of hDPCs, it is not unexpected that the levels of these odontogenesis-related markers changed considerably in miR-508-5p-treated cells. ALP, which expresses during the period of matrix deposition and maturation, is extensively used as an early marker of bone and tooth formation [32]. In contrast, DSPP, DMP-1, and OCN usually express at the terminal stage of odontoblast differentiation and dentinogenesis. Initially, DSPP was considered to be dentin specific. A later study reported the expression of DSPP in the bone with the amount of about 1/400 of that in dentin. As its predominant expression in dentin, DSPP remains a specific marker of odontoblastic differentiation [33]. For DMP-1, a major non-collagenous protein in the dentin matrix, its functions as a transcriptional factor to promote the expression of DSPP were reported, indicating that there might have a synergistic effect between DMP-1 and DSPP [34]. Similarly, osteocalcin is another member present in the dentin matrix and thought to be a reparative molecule [35, 36]. Obviously, it is the expression changes in these genes that underlie the molecular basis of miR-508-5p-induced effects on the differentiated potential of DPSCs.

## Conclusion

In summary, the present study demonstrated a regulatory role of miR-508-5p targeting GPNMB in the odontogenetic differentiation of hDPCs. Our findings provide further insights and new candidate target for the control of hDPC odontogenesis with potential therapeutic application in the treatment of dental diseases.

## Abbreviations

3′-UTR: 3′ Untranslated region
ALP: Alkaline phosphatase
DMP-1: Dentin matrix protein 1
DSPP: Dentin sialophosphoprotein
FACS: Fluorescence-activated cell sorting
GPNMB: Glycoprotein non-metastatic melanomal protein B
hDPCs: Human dental pulp stem cells
miR-508-5p: miRNA 508-5p
miRNAs: microRNAs
OCN: Osteocalcin
